# The therapeutic management of comorbid bipolar disorder and substance use disorder in female patients: our protocols and review of literature

**DOI:** 10.1192/j.eurpsy.2026.11095

**Published:** 2026-07-31

**Authors:** S. Boughdadi, B. El Hafidi, I. Adali, F. Manoudi

**Affiliations:** Psychiatry, Mohammed VI university hospital, Marrakech, Morocco

## Abstract

**Introduction:**

The comorbidity of bipolar disorder (BD) with substance use disorder (SUD) is one of the most frequent in the psychiatric field. This dual diagnosis leads to multiple challenges, in diagnostic, therapeutic and prognosis areas.

**Objectives:**

The aim of our study was to describe the therapeutic management of comorbid of BD and SUD in women hospitalized in the psychiatry department of Mohammed VI University Hospital in Marrakech; and compare it to the latest available literature.

**Methods:**

This was a retrospective study, it included female patients with comorbid BD and SUD, who were hospitalized in the psychiatry department of Mohammed VI University Hospital in Marrakech, between March 1^st^ 2022 and February 28^th^ 2023.

**Results:**

Our retrospective work included 36, out of which 14 had a comorbid SUD.The pharmacotherapeutic management of these patients focused on BD,it was based on monotherapy in 14.3% of cases.However, bi-therapy was more frequent,of mood stabilizers in 78.5% of cases: one antipsychotic (Olanzapine 57.1%) and one antiepileptic drug (Sodium valproate 42.8%,Carbamazepine 35.7%). In 7.1% of cases it was 2 antipsychotics (quetiapine + amisulpride). For most patients 85.7% there was an indication for complementary sedative treatment, the molecules used most were levomepromazine and chlorpromazine. Psychotherapy was recommended for the comorbid SUD but not started during hospitalization, patients were referred to the addiction center after their discharge from the hospital.

**Conclusions:**

In the management of comorbid BD and SUD, there are a few promising treatment methods, however all studies reported a lack of randomized clinical trials and proper research on the subject thus leading to a limited number of pharmacotherapy and even less psychosocial interventions. Our experience, although based on a limited sample, came to the conclusion that the management of patients in this situation is lacking, and calls for a more standardized evidence-based management that will take into account the particularities of both disorders and the specificities of each patient.

**Disclosure of Interest:**

None Declared

